# Supplementary material for: Regulation of CLK1 Isoform Expression by Alternative Splicing in Activated Human Monocytes Contributes to Activation-Associated TNF Production
Source: Cells. 2025 Dec 3;14(23):1925. doi: 10.3390/cells14231925 (PMC12691408; doi:10.3390/cells14231925)
Supplement: Supplementary file 1 [file cells-14-01925-s001.zip › cells-3988292-supplementary.pdf]

Supplementary

Table S1. RT-qPCR Primers.

Target	Primer name	Primer sequence
RPL13A	RPL13AFW	CCTGGAGGAGAAGAGGAAAGAGA
	RPL13ARV	TTGAGGACCTCTGTGTATTTGTCAA
B2M	B2MFW	ATGAGTATGCCTGGCCGTGTGA
	B2MRV	GGCATCTTCAAACCTCCATG
CLK1Total	CLK1TotalFW	AATGTGTGATAGCCATTATTTGGAA
	CLK1TotalRV	TTTGGCGATGTCCAGGTTCA
CLK1+4	CLK1+4FW	GTTCACATGGGAAGAGTCACC
	CLK1+4RV	TGCACTTAGTACGTCTCCACTC
CLK1Δ4	CLK1D4FW	CGTTCACATGGGAATGAAATTGT
	CLK1D4RV	GCTACATGTCTACCTCCCGC
TNFα	TNFαFW	CCCCAGGGACCTCTCTCTAA
	TNFαRV	TGAGGTACAGGCCCTCTGAT

Table S2. Antibodies.

#	Antibody name	Product number
1	GAPDH	MA5-15738
2	CLK1 antibody (AA 1-130)	ABIN7363161
3	CD14-PE/Cy7	Biolegend M5E2
4	TNFα-APC	Biolegend MAB11
5	eF506	Fisher eF506 Live Dead staining

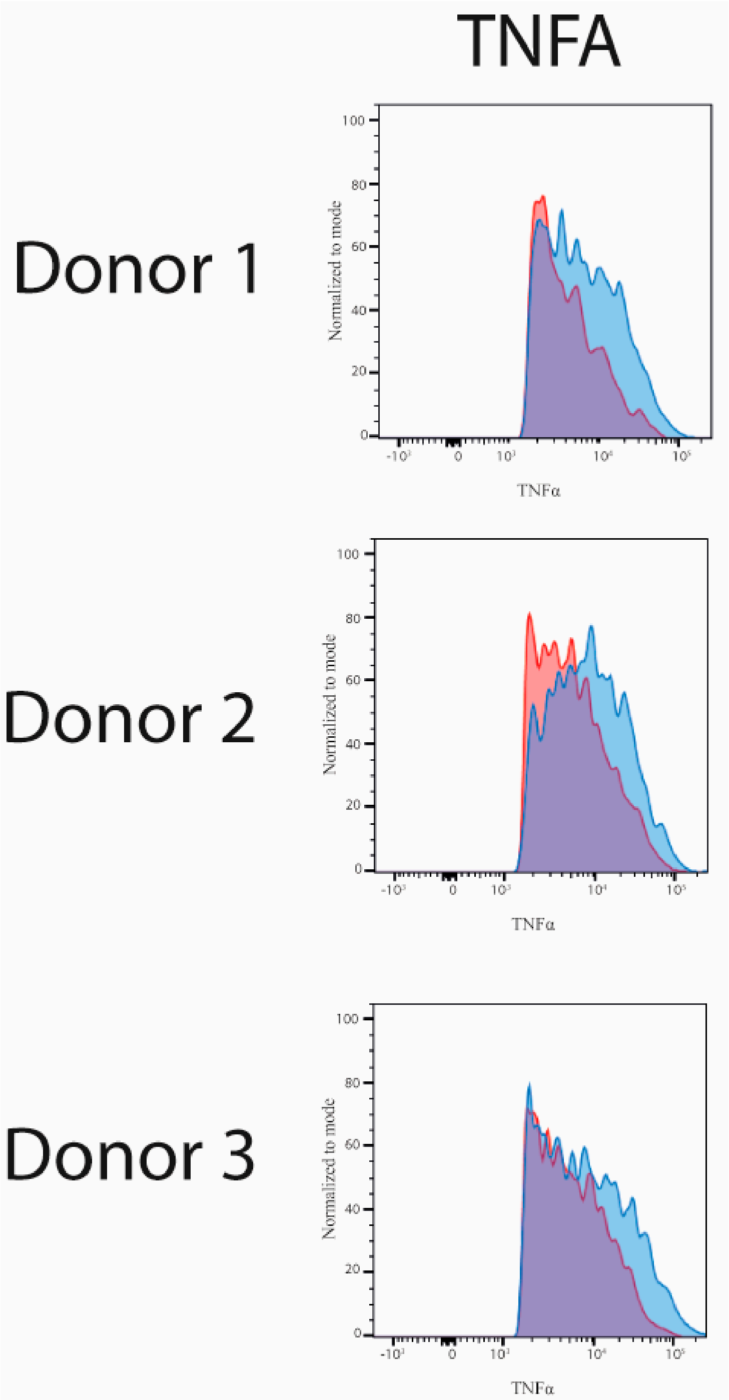

Figure S1. Flow cytometry CLK1 inhibition.

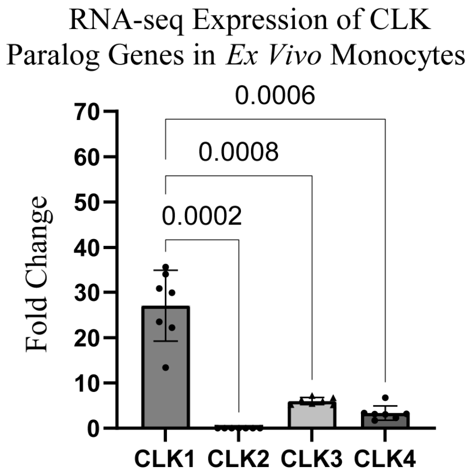

Figure S2. Expression of CLK paralogs in *ex vivo* monocytes measured by RNA-seq.
